# *Pneumocystis* Pneumonia in HIV-positive Adults, Malawi^1^

**DOI:** 10.3201/eid1302.060462

**Published:** 2007-02

**Authors:** Joep J.G. van Oosterhout, Miriam K. Laufer, M. Arantza Perez, Stephen M. Graham, Nelson Chimbiya, Phillip C. Thesing, Miriam J. Álvarez-Martinez, Paul E. Wilson, Maganizo Chagomerana, Eduard E. Zijlstra, Terrie E. Taylor, Christopher V. Plowe, Steven R. Meshnick

**Affiliations:** * University of Malawi, Blantyre, Malawi;; † University of Maryland School of Medicine, Baltimore, Maryland, USA; ‡ University of North Carolina, Chapel Hill, North Carolina, USA; § Michigan State University, East Lansing, Michigan, USA

**Keywords:** Pneumocystis pneumonia, HIV, Malawi, PCR, incidence, diagnosis, dispatch

## Abstract

In a prospective study of 660 HIV-positive Malawian adults, we diagnosed *Pneumocystis jirovecii* pneumonia (PcP) using clinical features, induced sputum for immunofluorescent staining, real-time PCR, and posttreatment follow-up. PcP incidence was highest in patients with the lowest CD4 counts but uncommon compared with incidences of pulmonary tuberculosis and bacterial pneumonia.

The incidence of *Pneumocystis jirovecii* pneumonia (PcP) in HIV-infected adults in the sub-Saharan African region remains uncertain. That PcP is common in African children <1 year of age is well documented (*1*), but reported prevalence and incidence rates in adult African populations vary widely (*2*). Many of these reports were cross-sectional studies in selected populations from tertiary hospitals (*3*–*5*), and therefore might contain selection bias that favors identifying higher rates of PcP.

To our knowledge, no large prospective studies have been done using broncho-alveolar lavage (BAL) in combination with immunofluorescent (IF) staining for *P. jirovecii* cysts, the diagnostic procedures of choice. Real-time PCR performed on sputum samples has high sensitivity but low specificity for PcP (*6*,*7*). The few studies in African adults that used PCR assays for *Pneumocystis* did not distinguish subclinical colonization from infection, mainly because of limited follow-up after diagnosis (*3*,*4*). We describe here the incidence of PcP from a large cohort study of HIV-infected Malawian adults that used a comprehensive diagnostic approach that included induced sputum with IF staining, real-time PCR, and follow-up after diagnosis and treatment.

## The Study

HIV-infected adults (>15 years of age), who sought treatment at a government health center in the township of Ndirande, Blantyre, Malawi, were enrolled in a prospective, community-based study to determine the incidence of infections that were preventable by trimethoprim-sulfamethoxazole prophylaxis (*8*). Clinical evaluations were performed monthly and at sick visits occurring between the scheduled monthly evaluations. CD4 counts were determined every 4 months. Standardized diagnostic and treatment guidelines and case definitions were used. At the time of the study, in Malawi, antiretroviral therapy (ART) was rarely used, and trimethoprim-sulfamethoxazole prophylaxis was not recommended.

Cases of suspected PcP were identified by patients’ clinical signs and symptoms, chest x-ray results, oxygen desaturation exercise test results (*9*), CD4 count, and failure to improve with antimicrobial treatment without activity against *P. jirovecii*. Patients’ sputum production was induced by an ultrasonic nebulizer with hypertonic saline, followed by IF staining for *P. jirovecii* cysts. A case was classified as clinical PcP when the IF staining for *P. jirovecii* cysts was positive or the participant had strong clinical evidence of PcP and negative IF. Clinical follow-up data were collected after the episode of suspected PcP.

After the study, real-time PCR for the *P. jirovecii* dihydropteroate synthase and human RNAase P (control DNA) was performed on DNA extracted from the stored induced sputum slides (*10*). Clinicians were not aware of the PCR results during the study, and laboratory staff performing the PCR was blinded to clinical information and IF results. A final diagnosis of confirmed PcP was made for any episode with a positive IF result, positive PCR result, or both, unless recovery (defined as resolution of respiratory symptoms present at the start of the episode) without PcP treatment was observed with a minimum of 4 weeks of follow-up. If the PCR results were positive but the patient recovered without active treatment against PcP, the result was interpreted as *Pneumocystis* colonization. A negative PCR result ruled out PcP diagnosis in patients who had received PcP treatment on the basis of clinical evidence alone.

Incidence rates of respiratory diagnoses per 100 person-years of follow-up were calculated with 95% confidence intervals (CIs) based on Poisson distribution. First and subsequent episodes in the same person were counted separately, except for PcP, because patients with PcP received secondary prophylaxis and exited the study. The CD4 count at the time of the episode or within the previous 6 months was used for analysis.

We used χ^2^, Mann-Whitney, and Student *t* tests for analysis of age, sex, and CD4 counts among diagnoses, respectively, using SPSS version 12 software (SPSS Inc., Chicago, IL, USA). The study was approved by the Institutional Review Boards of the University of Malawi College of Medicine, the University of Maryland, and Michigan State University.

Beginning in September 2002, 660 adults were enrolled in the study and followed up through August 2004. Baseline CD4 and World Health Organization stage data are shown in Table 1. Mean age was 31.7 years (range 16–66); 437 (66%) were female. Mean duration of follow up was 10.7 months (95% CI 10.4–11.5) per person. Eighty-six (13%) participants died, and 37 (6%) were withdrawn from the study because they started lifelong trimethoprim-sulfamethoxazole prophylaxis. Sixty-three participants (9.5%) left the area, 20 (3%) withdrew consent, and 119 (17%) were lost to follow up. A smaller proportion of patients from the low CD4 stratum exited the study than from the high CD4 content group.

**Table 1:** Baseline characteristics of study participants at enrollment, Malawi, 2002–2004*

Characteristic	No. episodes	PYO
CD4 (cells/mm^3^)		
0–99	125	83
100–199	159	145
200–499	271	268
>500	77	75
Missing at enrollment	28	19
HIV clinical stage†		
I	267	255
II	191	190
III	160	120
IV	42	25
Total	660	591

*PYO, person-years of observation (based on enrollment characteristics). †As defined by World Health Organization criteria.

Ninety-five episodes of suspected PcP occurred in 75 persons. Outcomes of these episodes are given in Table 2. A final diagnosis of confirmed PcP was made in 6 episodes, and 9 episodes of *Pneumocystis* colonization were recorded, with a mean follow up of 26 weeks (range 4–48 weeks). Table 3 shows the incidence rates of PcP and other respiratory conditions in the cohort.

**Table 2:** Outcomes in 95 episodes of suspected *Pneumocystis* pneumonia (PcP), Malawi, 2002–2004*

Final diagnosis†	Clinical diagnosis‡	No. episodes	IF	PCR	Follow-up data
Confirmed PcP	PcP	2	Pos	Pos	–
Confirmed PcP	PcP	1	Neg	Pos	–
Confirmed PcP	PcP	1	–	–	Death after 2 wk of PcP treatment (IS not done due to respiratory distress)
Confirmed PcP	Bronchiectasies	1	Neg	Pos	Death 1 wk after IS
Confirmed PcP	Tuberculosis	1	Neg	Pos	Improvement but short follow-up (2 wk)
*Pneumocystis* colonization/pulmonary KS	Pulmonary KS	3§	Neg	Pos	Death 23 wk after first IS
*Pneumocystis* colonization/tuberculosis	Tuberculosis	3	Neg	Pos	Recovery
*Pneumocystis* colonization/bacterial pneumonia	Bacterial pneumonia	2	Neg	Pos	Recovery
*Pneumocystis* colonization/unspecified respiratory illness	Unspecified respiratory illness	2	Neg	Pos	Recovery
*Pneumocystis* colonization/other diagnosis	Other diagnosis¶	1	Neg	Pos	Recovery
Unspecified respiratory illness	PcP	3	Neg	Neg	–
Tuberculosis	Tuberculosis	19	Neg	Neg	–
Bacterial pneumonia	Bacterial pneumonia	10	Neg	Neg	–
Unspecified respiratory illness	Unspecified respiratory illness	20	Neg	Neg	–
Unspecified respiratory illness	Unspecified respiratory illness	1	Neg	NA	Recovery (18 wk follow-up)
Other diagnoses¶	Other diagnoses¶	24	Neg	Neg	–
Other diagnosis¶	Other diagnosis¶	1	Neg	NA	Recovery (>1 y follow-up)

*IF, immunofluorescence stain; pos, positive; neg, negative; IS, induced sputum procedure; KS, Kaposi sarcoma. †Diagnosis based on clinical evidence, IF, and PCR from an IS sample, and follow-up after episode. ^‡^Diagnosis based on clinical evidence and IF from an IS sample. §Three episodes occurring in 1 person. ¶Among other diagnoses were sepsis, bronchitis, emphysema, pulmonary KS, and bronchiectasis.

**Table 3:** Incidence of *Pneumocystis* pneumonia (PcP) and other respiratory illnesses, Malawi, 2002–2004*

Diagnosis	All CD4 counts		CD4 0–99/mm^3^		CD4 100–199/mm^3^	
	No. events	Incidence, % (95% CI)	No. events	Incidence, % (95% CI)	No. events	Incidence, % (95% CI)
Confirmed PcP	6	1.0 (0.3–2.2)	5	5.7 (1.9–13.4)	1	0.6 (0.01–3.8)
Bacterial pneumonia†	102	17.3 (14.1–21.0)	35	40.2 (28.0–56.0)	42	28.6 (20.6–38.6)
Pulmonary tuberculosis	51	8.6 (6.4–11.3)	20	23.0 (14.0–35.5)	25	17.0 (11.0–25.1)
Unspecified respiratory illness	127	21.5 (17.9–25.6)	46	52.9 (38.7–70.5)	38	25.9 (18.3–35.5)

*CI, confidence interval. †Diagnosis based on new consolidations on a chest x–ray and response to antimicrobial drugs; includes cases with and without positive blood cultures.

With full diagnostic workup including posttreatment follow up as the gold standard for the diagnosis of PcP, the sensitivity of PCR alone was 100%, the specificity 88%, and the positive predictive value 31%. Among episodes in which PcP was suspected, the mean CD4 count in patients with confirmed PcP cases (42.5 cells/mm^3^, range 1–103) was not significantly lower than in those with *Pneumocystis* colonization (89.1 cells/mm^3^, range 7–194; p = 0.28), but was significantly lower than in those with other diagnoses (97.0 cells/mm^3^, range 1–311; p = 0.03). Mean age and sex distribution of confirmed PcP, *Pneumocystis* colonization, and other diagnoses were not significantly different. The case-fatality rate of confirmed PcP was 50%.

## Conclusions

This is the first community-based prospective study of PcP in a developing country. We found an incidence of PcP in Malawian HIV-infected adults of 1.0/100 person-years, similar to the rates observed in studies that used less comprehensive diagnostic approaches in South African miners (0.5/100 person-years) (*12*) and the placebo arms of trials of trimethoprim-sulfamethoxazole prophylaxis in Côte d’Ivoire (*13*,*14*). The incidence in persons with CD4 counts <200/mm^3^ (2.5/100 person-years) was clearly lower than in AIDS patients in the United States before the introduction of routine trimethoprim-sulfamethoxazole prophylaxis and highly active ART (10/100 person-years [*11*]). In the lowest CD4 count range (<100/mm^3^), PcP was common, although the incidence was low compared with that of bacterial pneumonia and pulmonary tuberculosis.

We believe it is unlikely that we missed many PcP cases among other diagnoses or losses to follow-up because of the intensive active and passive follow-up and because our facility provided expeditious, high-quality care free of charge. Allowing for reduced sensitivity of induced sputum compared to BAL (*7*) and considering cases with diagnostic uncertainty as PcP cases would still leave the PcP incidence low in the HIV-infected population in general.

We found that *Pneumocystis* colonization and confirmed PcP were equally common among patients with suspected PcP. More sensitive molecular detection methods would possibly have detected higher rates of colonization. It remains uncertain why certain HIV-infected persons clear *Pneumocystis* colonization while others develop PcP. The level of immune suppression as indicated by the CD4 count is a possible explanation, although our data do not support this. Genetic differences between *P. jirovecii* strains may be relevant (*15*). Variation in worldwide distribution of strains, as well as differences in host genetics and shorter survival in patients with a low range of CD4 counts, are possible causes of the lower PcP incidence in Africa than in developed countries.

The incidence of PcP in HIV-infected Malawian adults, diagnosed clinically and confirmed with molecular analysis, was low compared with the incidence of bacterial pneumonia and pulmonary tuberculosis at all levels of immunosuppression. PcP rarely occurred with CD4 cell counts >100 mm^3^. Among the most immunocompromised patients, PcP is an important diagnostic consideration.

## References

[1] Graham SM. HIV and respiratory infections in children. Curr Opin Pulm Med. 2003;9:215–20. 10.1097/00063198-200305000-00010 12682567

[2] Fisk DT, Meshnick S, Kazanjian PH. *Pneumocystis carinii* pneumonia in patients in the developing world who have acquired immunodeficiency syndrome. Clin Infect Dis. 2003;36:70–8. 10.1086/344951 12491205

[3] Hargreaves NJ, Kadzakumanja O, Phiri S, Lee CH, Tang X, Salaniponi FM, *Pneumocystis carinii* pneumonia in patients being registered for smear-negative pulmonary tuberculosis in Malawi. Trans R Soc Trop Med Hyg. 2001;95:402–8. 10.1016/S0035-9203(01)90197-X 11579884

[4] Aderaye G, Bruchfeld J, Olsson M, Lindquist L. Occurrence of *Pneumocystis carinii* in HIV-positive patients with suspected pulmonary tuberculosis in Ethiopia. AIDS. 2003;17:435–40. 10.1097/00002030-200302140-00018 12556698

[5] Chakaya JM, Bii C, Ng′ang′a L, Amukoye E, Ouko T, Muita L, *Pneumocystis carinii* pneumonia in HIV/AIDS patients at an urban district hospital in Kenya. East Afr Med J. 2003;80:30–5. 12755239 10.4314/eamj.v80i1.8663

[6] Maskell NA, Waine DJ, Lindley A, Pepperell JC, Wakefield AE, Miller RF, Asymptomatic carriage of *Pneumocystis jiroveci* in subjects undergoing bronchoscopy: a prospective study. Thorax. 2003;58:594–7. 10.1136/thorax.58.7.594 12832674 PMC1746733

[7] Cruciani M, Marcati P, Malena M, Bosco O, Serpelloni G, Mengoli C. Meta-analysis of diagnostic procedures for *Pneumocystis carinii* pneumonia in HIV-1–infected patients. Eur Respir J. 2002;20:982–9. 10.1183/09031936.02.01372002 12412693

[8] van Oosterhout JJG, Laufer MK, Graham SM, Thumba F, Perez MA, Chimbiya N, A community-based study of the incidence of trimethoprim-sulfamethoxazole-preventable infections in Malawian adults living with HIV. J Acquir Immune Defic Syndr. 2005;39:626–31. 16044018

[9] Smith DE, McLuckie A, Wyatt J, Gazzard B. Severe exercise hypoxaemia with normal or near normal X-rays: a feature of *Pneumocystis carinii* infection. Lancet. 1988;2:1049–51. 10.1016/S0140-6736(88)90066-9 2903279

[10] Alvarez-Martinez MJ, Miro JM, Valls ME, Moreno A, Rivas PV, Sole M, ; Spanish PCP Working Group. Sensitivity and specificity of nested and real-time PCR for the detection of *Pneumocystis jiroveci* in clinical specimens. Diagn Microbiol Infect Dis. 2006;56:153–60. Epub 2006 May 4. 10.1016/j.diagmicrobio.2006.03.010 16678378

[11] Kaplan JE, Hanson D, Dworkin MS, Frederick T, Bertolli J, Lindegren ML, Epidemiology of human immunodeficiency virus–associated opportunistic infections in the United States in the era of highly active antiretroviral therapy. Clin Infect Dis. 2000;30:S5–14. 10.1086/313843 10770911

[12] Corbett EL, Churchyard GJ, Charalambos S, Samb B, Moloi V, Clayton TC, Morbidity and mortality in South African gold miners: impact of untreated disease due to human immunodeficiency virus. Clin Infect Dis. 2002;34:1251–8. 10.1086/339540 11941552

[13] Wiktor SZ, Sassan-Morokro M, Grant AD, Abouya L, Karon JM, Maurice C, Efficacy of trimethoprim-sulphamethoxazole prophylaxis to decrease morbidity and mortality in HIV-1–infected patients with tuberculosis in Abidjan, Cote d'Ivoire: a randomised controlled trial. Lancet. 1999;353:1469–75. 10.1016/S0140-6736(99)03465-0 10232312

[14] Anglaret X, Chene G, Attia A, Toure S, Lafont S, Combe P, Early chemoprophylaxis with trimethoprim-sulphamethoxazole for HIV-1– infected adults in Abidjan, Cote d’Ivoire: a randomised trial. Cotrimo–CI Study Group. Lancet. 1999;353:1463–8. 10.1016/S0140-6736(98)07399-1 10232311

[15] Beard CB, Fox MR, Lawrence GG, Guarner J, Hanzlick RL, Huang L, Genetic differences in *Pneumocystis* isolates recovered from immunocompetent infants and from adults with AIDS: epidemiological implications. J Infect Dis. 2005;192:1815–8. Epub 2005 Oct 13. 10.1086/497381 16235182

